# Rationale and design of a smartphone‐enabled, home‐based exercise program in patients with symptomatic peripheral arterial disease: The smart step randomized trial

**DOI:** 10.1002/clc.23362

**Published:** 2020-04-23

**Authors:** Arash Harzand, Alexander A. Vakili, Alaaeddin Alrohaibani, Smah M. Abdelhamid, Neil F. Gordon, John Thiel, Jaime Benarroch‐Gampel, Victoria J. Teodorescu, Keri Minton, Nanette K. Wenger, Ravi R. Rajani, Amit J. Shah

**Affiliations:** ^1^ Division of Cardiology, Department of Medicine Emory University School of Medicine Atlanta Georgia USA; ^2^ Atlanta Veterans Affairs Medical Center Decatur Georgia; ^3^ Department of Medicine Emory University School of Medicine Atlanta Georgia USA; ^4^ INTERVENT International Savannah Georgia USA; ^5^ Centre for Exercise Science and Sports Medicine, School of Therapeutic Sciences University of the Witwatersrand Johannesburg South Africa; ^6^ Division of Vascular Surgery, Department of Surgery Emory University School of Medicine Atlanta Georgia USA; ^7^ Grady Memorial Hospital Atlanta Georgia USA; ^8^ Department of Epidemiology, Rollins School of Public Health Emory University Atlanta Georgia USA

## Abstract

**Background:**

Supervised exercise therapy (SET) is recommended in patients with symptomatic peripheral arterial disease (PAD) as first‐line therapy, although patient adoption remains low. Home‐based exercise therapy (HBET) delivered through smartphones may expand access. The feasibility of such programs, especially in low‐resource settings, remains unknown.

**Methods:**

Smart Step is a pilot randomized trial of smartphone‐enabled HBET vs walking advice in patients with symptomatic PAD in an inner‐city hospital. Participants receive a smartphone app with daily exercise reminders and educational content. A trained coach performs weekly phone‐based coaching sessions. All participants receive a Fitbit Charge HR 2 to measure physical activity. The primary outcome changes in 6‐minute walking test (6MWT) distance at 12 weeks over baseline. Secondary outcomes are the degree of engagement with the smartphone app and changes in health behaviors and quality of life scores after 12 weeks and 1 year.

**Results:**

A total of 15 patients are randomized as of December 15, 2019 with a mean (SD) age of 66.1 (5.8) years. The majority are female (60%) and black (87%). At baseline, the mean (SD) ABI and 6MWT were 0.86 (0.29) and 363.5 m, respectively. Enrollment is expected to continue until December 2020 to achieve a target size of 50 participants.

**Conclusions:**

The potential significance of this trial will be to provide preliminary evidence of a home‐based, “mobile‐first” approach for delivering a structured exercise rehabilitation program. Smartphone‐enabled HBET can be potentially more accessible than center‐based programs, and if proven effective, may have a potential widespread public health benefit.

## 1 INTRODUCTION

An estimated 8.5 million Americans (or 7.2% of the US adult population) are estimated to have peripheral arterial disease (PAD), defined as an ankle‐brachial index (ABI) of <0.9.^1^ Nearly, 10% of PAD patients are symptomatic with claudication—a reproducible leg pain with ambulation that is relieved with rest.^2^ Following the onset of symptoms, patients with PAD are at an increased risk of adverse outcomes including worsening functional status, amputation, myocardial infarction, stroke, and death.^1^, ^3^ African Americans in particular are at an increased risk of PAD and experience greater walking impairment and more severe disease than non‐Hispanic white individuals.^4^, ^5^

Supervised exercise therapy (SET), a 12‐week program with multiple in‐person weekly sessions, is recommended as a first‐line therapy in symptomatic PAD.^6^ Current guidelines for SET include that participants perform intermittent bouts of treadmill‐based walking exercise at least three times per week. During each session, patients are advised to walk in intervals with an intensity level to induce moderate to moderately‐severe leg symptoms within 5 to 10 minutes, at which point they should rest until the pain dissipates. Walking intervals should be repeated with a goal of accumulating up to 60 minutes of total exercise time during each session.^7^ Participation in SET has been shown to improve walking ability, overall functional status, and health‐related quality of life (QoL) in symptomatic PAD, and is considered first‐line therapy with a Class I recommendation from established practice guidelines in patients with claudication.^8^, ^9^

Despite the benefits, SET remains significantly underutilized among eligible patients.^10^, ^11^ In a recent meta‐analysis, less than 25% of patients with claudication enrolled in SET.^10^ Participation in SET has been limited by a lack program availability, poor reimbursement, inconvenience, and patient motivation.^12^ Even despite a National Coverage Determination by the Centers of Medicare and Medicaid Services in 2017 for SET reimbursement, the availability and uptake of SET programs have remained exceedingly low.^13^ This suggests that SET may remain an unlikely option for many patients, especially in low‐resource, public‐hospital settings where third‐party payer coverage for healthcare services is limited.

Home‐based exercise therapy (HBET) has been proposed as an alternative for patients with limited access to facility‐based SET.^6^, ^14^ Structured, home‐based programs have the potential to be a more convenient and acceptable option for patients than supervised exercise.^14^ Although several randomized trials have documented improved walking ability and QoL from HBET over usual care, more recent trials have failed to confirm these findings.^15^, ^16^, ^17^, ^18^, ^19^

### 1.1 Rationale for the smart step study

Smartphone ownership is on the rise^20^ and new opportunities exist to efficiently provide services with wider accessibility without increases in personnel and capital resources. Smartphones have been used to successfully deliver home‐based cardiac rehabilitation (CR), a program similar in broad scope and design to HBET for PAD, with high adherence and low attrition.^21^, ^22^ Although several ongoing studies are also evaluating smartphones for various exercise applications in PAD,^23^, ^24^ Smart Step is the first study, to our knowledge, to assess the potential for smartphones to deliver HBET in a low resource, public hospital setting with an economically disadvantaged population. We recently showed the feasibility of smartphone‐enabled CR within the Department of Veterans Affairs with high adherence (90%) and satisfaction (80%) and a 20% gain in functional status.^25^ Based on knowledge gained from this endeavor, we designed the Smart Step Study to help PAD patients overcome similar barriers to SET participation.

### 1.2 Hypotheses and objectives

The overarching goal of the Smart Step Study is to evaluate the feasibility of a smartphone‐enabled, home‐based exercise program for PAD in a low‐resource setting where access to a traditional, facility‐based SET program is essentially absent. The primary outcome of interest is functional improvement after 12‐weeks of enrollment. We will also assess for participant engagement with the smartphone app and phone‐based coaching. If successful, this study will generate novel information on how PAD patients in economically disadvantaged settings respond to a smartphone‐based exercise program and provide evidence to proceed with larger trials to determine efficacy.

## 2 METHODS

### 2.1 Trial design and oversight

**FIGURE 1:**
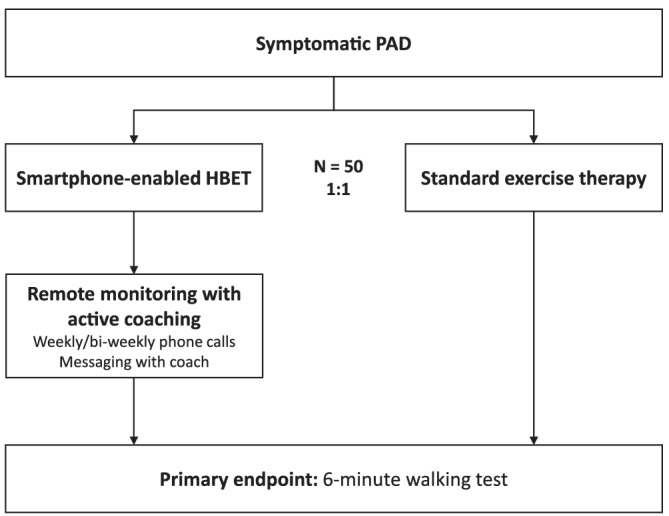
Smart step study design. Data as of December 2019

The Smart Step Study (ClinicalTrials.gov NCT03479255) is a single‐site, prospective, open‐label, pilot randomized trial of patients with symptomatic PAD who are eligible for SET. The study design is illustrated in Figure 1. Eligible subjects are randomized to either a smartphone‐enabled HBET program or to walking advice with standard exercise guidance. Potential participants are recruited from the vascular surgery and cardiology clinics at Grady Memorial Hospital, a tertiary academic medical center and public benefit hospital in downtown Atlanta, GA, and screened for eligibility. Eligible participants are then randomized in a 1:1 ratio using a random number generator. Women are oversampled to achieve a 50% target representation. The study protocol has been approved by the Institutional Review Board at Emory University and the Research Oversight Committee at Grady Memorial Hospital.

The study was designed by the Executive Committee which includes three cardiologists and three vascular surgeons. Funding was provided by the Emory Woodruff Health Sciences Center. The industry partners (Moving Analytics and INTERVENT) were not involved in the study design and did not provide funding but are participating in data collection. The Clinical Endpoint and Data Monitoring Committees are located at Emory University.

### 2.2 Eligibility criteria

Subjects aged 18 to 89 years with evidence of PAD based on a resting ABI ≤0.90 and clinically stable intermittent claudication as determined by the referring physician are eligible. All participants must have a personal Android (Google, Mountain View, California) or iOS (Apple, Cupertino, California) smartphone in working condition and be able to provide informed consent. Those with both typical and atypical symptoms of claudication are also considered, as prior studies have demonstrated that patients with atypical symptoms also have significant functional limitation that improves significantly with supervised exercise.^26^, ^27^, ^28^, ^29^ Patients are excluded if they have any life‐threatening conditions (eg, sepsis, unstable angina, or New York Heart Association [NYHA] class IV heart failure), critical limb ischemia, or a condition other than PAD that causes limitation in walking before the onset of claudication. Detailed inclusion and exclusion criteria with justifications are listed in Table 1.

**TABLE 1:** Inclusion and exclusion criteria

Inclusion criteria
Age 18‐89 years Able to provide informed consent Clinically stable intermittent claudication Diagnosis of peripheral arterial disease (PAD) with one of the following: Ankle‐brachial index (ABI) ≤ 0.9 or ABI > 0.9 but ≤1.00 at rest with 20% drop in ABI with exercise or heel‐rise test or ABI > 0.9 with other evidence of lower extremity PAD such as a positive anatomic study (ie, ultrasound, computed tomography or invasive angiography) or prior lower extremity revascularization (surgical or endovascular) for PAD or ABI > 1.3 with additional evidence of PAD (ie, pulse volume recording [PVR], toe‐brachial index or arterial duplex studies). Possess a working Android or iOS smartphone to install the Movn app

Exclusion criteria	Justification
1. Individuals whose walking is limited by a condition other than PAD (eg, severe angina or dyspnea, arthritis, muscle weakness or pain).	Intervention is designed to improve PAD related walking impairment.
2. Any life‐threatening process including (eg, sepsis, critical limb ischemia [Rutherford class 4‐6], unstable angina, active malignancy with life expectancy <6 months, severe New York Heart Association [NYHA] Class IV heart failure)	Exercise may be potentially unsafe for these patients.
3. Active behavioral conditions such as uncontrolled schizophrenia or illicit drug addiction that, in the opinion of the study team, will interfere with active participation.	May interfere with ability to fully engage in the study.

### 2.3 Study procedures and follow‐up

Upon enrollment, participants are randomly assigned to either the smartphone‐enabled HBET or usual care arm and followed prospectively for 12 weeks. Regardless of the treatment arm, all subjects receive a Fitbit Charge 2 (Fitbit, San Francisco, California) to record various measures of physical activity including steps, exercise activity, and heart rate (HR). All participants are then prescribed an exercise plan according to their study arm as outlined below.

#### 2.3.1 Standard exercise therapy

Patients randomized to the usual care arm receive counseling by their physician to perform self‐monitored walking exercise according to established practice guidelines for PAD. This represents current standard practice at Grady Memorial Hospital and includes a recommendation to perform between 30‐ and 45‐minutes walking exercise three times per week. This is based on prior meta‐analyses which have found that walking at least 30 minutes is more beneficial than shorter durations, and that benefit appears to peak at 45 minutes.^9^, ^30^, ^31^ Participants are instructed to walk at an intensity that induces onset of claudication within 3 to 5 minutes and moderate to moderately severe claudication within 8 to 10 minutes, at which point they should stop and rest until their claudication subsides. After their symptoms dissipate, they should resume this same cycle of exercise until their target exercise duration is reached. In addition to exercise, participants in the standard exercise group receive counseling on healthy lifestyle behaviors including dietary changes, medication adherence, and smoking cessation if needed.

#### 2.3.2 Smartphone‐enabled exercise therapy

**FIGURE 2:**
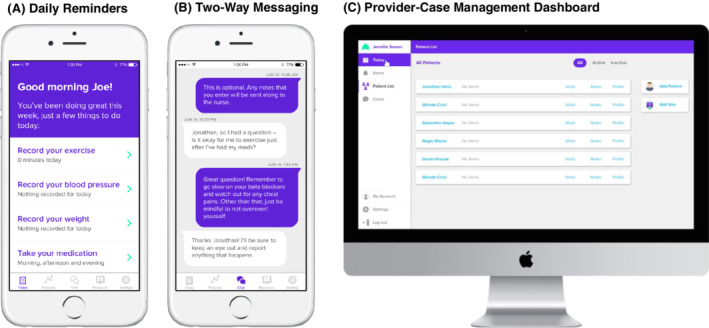
Movn smartphone app and dashboard

Patients in the smartphone‐enabled arm receive an app (Movn, Moving Analytics, Los Angeles, California) to guide their exercise program in addition to the fitness tracker which also synchronizes with the app. Our study team has successfully piloted the use of Movn in the delivery of home‐based CR to veterans with ischemic heart disease.^25^ Movn is a commercially available home‐based exercise rehabilitation program based on the MULTIFIT,^32^ a case‐management system for secondary prevention and patient surveillance after acute MI developed by investigators at Stanford University School of Medicine and implemented within Kaiser Permanente of Northern California. In previous studies, MULTIFIT has been shown to significantly improve low‐density lipoprotein cholesterol (LDL‐C) levels, functional capacity, and rates of smoking cessation after 12 weeks of enrollment compared to usual care.^32^, ^33^ Movn provides virtual delivery of MULTIFIT through a patient‐facing smartphone app that synchronizes with a provider‐facing online case‐management dashboard to allow for remote monitoring of participants' progress (Figure 2). The app includes a virtual diary for participants to enter data on exercise sessions (including exercise time, frequency and symptoms), blood pressure (BP), HR, weight and medication adherence, as well as provide exercise reminder notifications, educational material on heart and vascular health, and two‐way secure messaging with a health coach. The coach is an exercise physiologist with extensive experience in remote coaching (via INTERVENT, Savannah, Georgia). The initial coaching session occurs via video conference and subsequent sessions are performed with brief telephone calls lasting approximately 15 minutes in duration. Customized education modules designed by INTERVENT are used to specifically educate the participants on PAD. The coach facilitates each participant's exercise program by remotely monitoring their progress via the dashboard and providing feedback during scheduled telephone calls occurring at 1 to 2‐week intervals. Participants in the smartphone‐enabled arm receive the same educational topics and guideline‐directed exercise prescription as for the standard exercise arm.

### 2.4 Study measures

The primary outcome changes in 6‐minute walk distance between baseline and 12 weeks. Secondary outcomes include (a) changes in disease burden and health‐related QoL scores at 12 weeks and 1 year (b) changes in daily ambulatory activity measured by the Fitbit, and (c) degree of engagement with the app and coaching program. A full list of study measures along with timing of measurements are included in Table 2. There are no exploratory endpoints. All prespecified outcomes are obtained by trained study staff. Based on the nature of the intervention requiring logistical coordination with Moving Analytics and INTERVENT for treatment allocated patients, blinding was not considered feasible.

**TABLE 2:** Study measures

	Time point		
	Baseline	12 weeks	1 year
Primary endpoint			
6‐minute walk test distance	X	X	X
Secondary endpoints			
Walking Impairment Questionnaire	X	X	X
Short Form‐36 questionnaire	X	X	X
Smartphone app and Fitbit usage	X	X	X
Ambulatory activity (average daily steps)		X	X
Peripheral artery disease risk factors	X	X	X
Clinical endpoints (all‐cause emergency department and hospital admissions, critical limb ischemia, cardiovascular events including acute coronary syndromes and stenting, and all‐cause mortality)		X	X

#### 2.4.1 6‐minute walk test

The 6‐minute walk test (6MWT) distance is an objective and well‐validated measure of walking ability that predicts mobility loss and mortality in PAD with excellent test‐retest reliability, and is our primary endpoint.^12^, ^34^, ^35^ Distances walked between repeat 6MWTs by PAD patients have a high‐reliability coefficient (>0.90) and a low coefficient of variation (~10%).^34^, ^35^ The 6MWT offers several advantages over treadmill testing in PAD as it correlates more closely with physical activity levels in the community and is not associated with a learning effect when repeated testing is performed.^36^, ^37^ Participants perform a 6MWT by walking back and forth along a 100‐ft hallway using a standard protocol.^19^ The total distance walked, claudication onset time, defined as the time at which ambulation could not continue due to maximal pain, and the pain‐free walking distance, are recorded to quantify the severity of claudication. In a recent randomized study, the minimal clinically important difference (MCID) for 6MWT distance for small, moderate and large changes after 3 months of home‐exercise were 11, 28, and 45 m, respectively.^38^

#### 2.4.2 Survey data

Data from both the Walking Impairment Questionnaire (WIQ) and Short‐Form 36 (SF‐36) are collected as secondary outcomes. All surveys are self‐administered by the participant. The WIQ is a validated PAD‐specific measure of patient‐reported walking ability including walking speed and distance, and the ability to climb stairs.^39^ Each domain is scored on a scale of 0 to 100 with 0 representing the most significant limitation and 100 representing no difficulty. Changes in walking ability as a result of therapeutic interventions, such as exercise, have been correlated with improvements in the WIQ score.^18^, ^19^ The SF‐36 is a well‐validated QoL assessment that is frequently used to assess changes in response to therapeutic interventions in PAD, including trials of home exercise.^19^, ^40^ The SF‐36 includes eight health dimensions (eg, physical functioning, bodily pain, general health) which will be converted to a scale ranging from 0 (worst possible score) to 100 (best possible score).^40^

#### 2.4.3 Daily ambulatory activity

Daily ambulatory activity is assessed as a secondary endpoint using a wrist‐worn activity monitor (Fitbit Charge HR) which measures step counts, actigraphy, and resting HR. Fitbit devices have been shown to be more valid than other consumer activity monitors in measuring walking activity.^41^ Participants are instructed to wear the device daily during waking hours and remove it at night to recharge. The device is connected via Bluetooth to their personal smartphone device via the Fitbit app. The device records the number of steps taken and other data points on a minute‐to‐minute basis. With participants' consent, a member of the study team remotely accesses the participants' activity data using the Fitbit online dashboard.

#### 2.4.4 Ankle‐brachial index

All participants undergo measurement of the ABI at baseline to determine eligibility. The ABI is associated with the degree of functional impairment and the rate of functional decline among older adults in PAD.^28^ Since prior randomized trials of exercise therapy in PAD have not demonstrated an improvement in ABI after exercise, the ABI is not repeated after the 6MWT or during any follow‐up visit. After 10 minutes at rest, an ABI is obtained from the more severely diseased lower extremity using a standard protocol.^42^ Both the ankle and brachial systolic pressures are measured by Doppler. Measurements are taken from the posterior tibial and dorsalis pedis arteries at the ankle in both legs, and the higher of the arterial pressures from the more severely diseased legs is recorded as the resting ankle systolic pressure. Similarly, the higher systolic pressure is recorded as the brachial systolic pressure for the ABI, calculated as the ratio of the ankle systolic pressure to the brachial systolic pressure.

### 2.5 Statistical methods

#### 2.5.1 Key endpoints

Our primary outcome is change in 6MWT distance from baseline to 12 weeks (ie, end of program completion). All remaining outcomes are secondary and include survey data from the WIQ and SF‐36 questionnaires, changes in daily ambulatory activity from the Fitbit, modifiable PAD risk factors (such as blood pressure and smoking status), self‐reported exercise and medication adherence collected via the smartphone app, the degree of participant engagement with the app individual features (eg, chats, educational videos), and pre‐specified clinical endpoints (eg, emergency department visits, hospital admissions, critical limb ischemia, cardiovascular events, or death prior to program completion).

#### 2.5.2 Power and sample size calculations

We powered the study on the primary outcome of 6MWT distance. All other outcomes are secondary. In a recent study of HBET, the estimated effect size (ES) was a Cohen's d = 0.96 in the comparison of change in 6MWT distance between HBET and an attention control condition based on a sample size of 50 per group (mean change of 55 ± 57 m in the intervention group vs no change in the control group).^38^ Assuming an alpha of 0.05, 50 participants total (25 per group) provides 93% power to detect a similar effect. If we have a 32% dropout rate,^15^, ^19^, ^43^ the power decreases to 80%. If all 50 participants are retained, however, the minimum detectable difference between groups at 80% power is 45 m (SD 57 m). For reference, these are considered large effect sizes.^38^ Nonetheless, such differences have been seen in the literature and are considered justified.

#### 2.5.3 Statistical analysis

For analyses involving comparisons of groups we will perform two‐sample, independent *t*‐tests for normally distributed data, and logarithmic transformations for positive skewed distributions. For highly skewed data where log transformation does not adequately correct the skew, we will use non‐parametric rank‐order tests such as the Wilcoxon rank sum. For between‐group differences, 95% confidence intervals will be constructed, and multivariable adjustments using linear regression will be used where large group imbalances exist. Analyses will be performed as intention‐to‐treat, but we will also do sub‐group analyses based on the degree of engagement with the program. Multiple imputations will be used for missing data using established methods,^44^ and per‐protocol analyses will also be conducted. The a priori level for statistical significance was a two‐sided *P* < .05.

There are no sub‐studies planned, but we will perform predefined subgroup analyses to explore efficacy according to several baseline characteristics including age, ABI results, functional status, revascularization history, and degree of engagement with the smartphone app and coaching program.

## 3 RESULTS

As of December 15, 2019, a total of 35 patients have been screened and 15 participants have been enrolled and randomized. No significant differences were found between screened and enrolled participants. To date, 13 have completed the study (eight in the treatment group and seven in the control group). See Figure S1 in the Supplementary Appendix. Among all enrolled patients, the mean (SD) age was 66.1 (5.8) years, 9 (60%) were women, and 13 (87%) were black (Table 3). The mean (SD) ABI is 0.86 (0.29), reported as the worst diseased extremity. The baseline 6MWT distance among all participants was 363.5 m. As expected in PAD patients, there was a high prevalence of concomitant hypertension and dyslipidemia. Overall, the prevalence of coronary artery disease (CAD) was lower than expected at 26.7%. The population was well‐treated, however, with 73.3% of patients prescribed both an aspirin and statin.

**TABLE 3:** Baseline characteristics

Characteristic	N = 15
Age	66.1 (5.8)
Female	9 (60)
BMI	29.5 (5.9)
Current smoker	10 (66.7)
ABI	0.86 (0.29)
6MWT distance	363.5 (113.0)
Race	
Black or African American	13 (86.7)
White	2 (13.3)
Comorbidities	
Hypertension	13 (86.7)
Dyslipidemia	12 (80.0)
Diabetes mellitus	8 (53.3)
CAD	4 (26.7)
Chronic systolic HF	2 (13.3)
CVA or TIA	2 (13.3)
History of PCI	3 (0.20)
Medication	
Aspirin	11 (73.3)
Plavix	2 (13.3)
Statin	11 (73.3)
ACE or ARB	9 (60)
Beta‐blocker	7 (46.7)

*Note*: Values are N (%) or mean (SD).

Abbreviations: ABI, ankle‐brachial index; ACE, angiotensin‐converting enzyme; ARB, angiotensin‐receptor blocker; BMI, body mass index; CAD, coronary artery disease; CVA, cerebrovascular accident; HF, heart failure; PCI, percutaneous coronary intervention; TIA, transient ischemic attack; 6MWT, 6‐minute walking test.

## 4 DISCUSSION

The Smart Step Study was designed to answer an important question in the management of PAD patients: what the feasibility of a smartphone‐enabled program of remote exercise is to improve functional capacity in patients with claudication receiving care in a low resource healthcare setting. Although several studies are currently underway examining the role of smartphones in PAD management,^23^, ^45^ to our knowledge, the Smart Step study is the first study to evaluate a smartphone‐enabled HBET program in a low resource, public‐hospital setting with a majority of African American patients. The Smart Step trial also integrates a contemporary wearable activity monitor with the Fitbit Charge 2 which will complement that of several other trials which assessed the value of mobile technologies in the evaluation and management of symptomatic PAD.^17^, ^18^, ^19^, ^43^

The Smart Step Study is unique because it primarily targets patients with lower socioeconomic status (SES) in an inner‐city hospital. Although SET program availability is limited in general, PAD patients in underserved populations may have an even higher risk of poor outcomes due to lack of adequate insurance and reliance on lower‐resource healthcare systems without available SET programs. This increases the potential impact of Smart Step to fill a vital gap in care for such individuals, of which large numbers exist and healthcare costs are high. Additionally, since many lower income adults rely on a smartphone to access the internet, the program will likely be feasible in this group as well.^20^

Given the rising prevalence of PAD and the limited availability of supervised exercise, particularly in low resource settings, there is a mounting public health need to develop inexpensive, portable, and scalable SET delivery methods. Despite the substantial benefits associated with supervised treadmill walking,^8^, ^46^ many physicians do not recommend SET to patients with PAD and, even when referred, most eligible individuals do not enroll due to limited availability, lack of insurer coverage, and difficulty with transportation. In combination, these factors make participation in SET burdensome and impractical. The recent availability of smartphones and the proven feasibility of Movn, a smartphone‐enabled home‐based cardiac rehabilitation program, has led to new opportunities to address limitations of SET in a low resource environment with a modified version of the program tailored specifically for PAD.

The smartphone program specifically has several hallmark components that present an evolution in home‐based therapy. First, it automates the data transfer of physical activity (steps, flights of stairs, active minutes) from the patient to the provider, who views the data using a secured online dashboard. Furthermore, it serves as an electronic diary for the patient to record various activities, including medication use, smoking, and exercise sessions—all data are shared with the coach and allow the patient to both reflect on his/her own activity, as well as be accountable to the coach. The patient can also message the coach through the app (between phone sessions), which improves access to care. Coaches can be alerted to patients who have not logged exercise for an extended period, allowing for earlier detection of non‐compliance. Furthermore, the program is integrated with various wearable devices, which potentially allow for sharing HR, among other physiologic data. Education and surveys can also be delivered via the app and organized in the patient's electronic profile.

Prior studies of HBET using mobile technologies have primarily integrated wearable activity monitors as opposed to smartphones and have yielded conflicting results.^17^, ^18^, ^19^ Two early trials by Gardner et al demonstrated high rates of adherence with a quantified home exercise program using a step activity monitor that resulted in significant improvements in claudication symptoms and walking parameters similar to a supervised program.^17^, ^18^ In an early trial, 119 participants were randomized to HBET with an ankle‐mounted step activity monitor (StepWatch 3, Cyma Inc.), traditional supervised treadmill exercise, or usual care. Participants in the HBET group were instructed to exercise in a manner similar to a supervised program. After 12 weeks, both the HBET and supervised exercise groups significantly increased their claudication onset and peak walking times (COT and PWT, respectively) vs usual care. These findings were replicated in the subsequent NEXT Step Study by Gardner et al in 2014 that randomized 180 patients in a similar manner to either HBET, supervised exercise, or attention control.^18^ After 12‐weeks, participants in the home‐based program again demonstrated significant improvements in both COT (*P* < .001) and PWT (*P* < .001) as well as exercise capacity measured by 6‐MWT distance (*P* = .028) compared to usual care. Participants in the home‐based arm performed significantly more exercise measured in terms of total time (1011 vs 762 minutes; *P* = .009) and time per exercise session (38.3 vs 24.7 minutes; *P* < .001) despite a lack of direct supervision.

In contrast, the more recent HONOR Trial by McDermott et al of an HBET intervention using a waist‐worn pedometer (Fitbit Zip) yielded substantially different results. In HONOR, 200 patients with symptomatic PAD were randomized to HBET or to usual care with walking advice only.^19^ Participants in the home‐exercise group completed 12‐weeks of exercise, initially with weekly sessions at the hospital for 4 weeks followed by 8 weeks of completely home‐based exercise with telephone coaching and with the wearable monitor. After 9 months of follow‐up, there was no significant difference in 6‐minute walk distance between the home‐based intervention and usual care group with a surprising trend towards better performance among participants in the usual care group who did not receive exercise coaching (5.5 vs 14.4 m; 95% CI, −26.0 to 8.2; *P* = .31). Total step counts from the Fitbit's accelerometer did not statistically differ between the two groups.

The Smart Step Study offers several important additions to these and other prior HBET studies. First, whereas prior studies have used passive activity trackers such as pedometers and actigraphs only, the use of a smartphone app to enable self‐management and provide clinical support is a novel addition which has only been recently explored in PAD management,^23^, ^45^ and to our knowledge has not been previously deployed in a low‐resource hospital setting. The addition of Movn and INTERVENT also offer novel additions in smartphone‐based delivery and coach training. The study coach underwent rigorous coach training by INTERVENT which has extensive experience in behaviorally‐oriented telephonic health coaching in patients with ischemic heart disease and stroke.^47^

## 5 CONCLUSION

Access to a structured exercise walking program is of paramount importance to patients with PAD; however, the multiple barriers including limited availability, inconvenience, and cost has made delivery of facility‐based SET programs challenging. A smartphone‐enabled HBET program, if effective, could have far‐reaching implications for the growing number of PAD patients especially in lower resource settings where participation in SET is virtually nonexistent.

## CONFLICT OF INTEREST

Dr. Harzand reports serving on a science advisory board for Moving Analytics. Dr. Gordon is the managing member of a population health management company, INTERVENT International. None of the remaining authors report any potential conflicts of interest. The decision to publish was made solely by the authors.

## Supporting information


**Appendix** **S1:** Supporting information

**Figure S1** Study Enrollment Chart. Data as of December 2019.
