# Supplementary material for: Rationale and design of a smartphone‐enabled, home‐based exercise program in patients with symptomatic peripheral arterial disease: The smart step randomized trial
Source: Clin Cardiol. 2020 Apr 23;43(6):537–45. doi: 10.1002/clc.23362 (PMC7298994; doi:10.1002/clc.23362)
Supplement: Supplementary file 1 — Appendix S1: Supporting information [file CLC-43-537-s001.docx]

**APPENDIX**

**Executive Committee**

Amit J. Shah, MD, MPH (Co-Chair and Co-Principal Investigator); Ravi Rajani, MD (Co-Chair and Co-Principal Investigator); Nanette K. Wenger, MD (Co-Chair and Co-Principal Investigator); Arash Harzand, MD, MBA; Jaime Benarroch-Gampel, MD, MS; Victoria Teodorescu, MD

**Data Monitoring Committee**

Charles D. Searles (Chair), Modele O. Ogunniyi, Xiangqin Cui (statistician)

**Clinical Endpoint Committee**

Emory University members:
Arash Harzand (Chair), Amit J. Shah

**Moving Analytics**

Nancy Miller-Houston (Clinical Scientist), Lynda Murdock (Programming), Harsh Vathsangam (Study Management)

**INTERVENT**

Neil Gordon (Study Physician); John Thiel (Clinical Coach)

**Supplementary Figure Legends**

Figure S1. Study Enrollment Chart. Data as of December 2019.
